# Coagulopathie aigue précoce des traumatismes crâniens graves: mortalité et facteurs pronostiques

**DOI:** 10.11604/pamj.2014.17.107.2833

**Published:** 2014-02-13

**Authors:** Abdelhamid Hachimi, Mina Elkhayari, Ibtissam Chaibi, Rachid Razine, Amra Ziadi, Mohamed Abdenasser Samkaoui

**Affiliations:** 1 Service de Réanimation Polyvalente, CHU Mohammed VI, Faculté de Médecine et de Pharmacie, Université Cadi Ayyad, Marrakech, Maroc; 2 Laboratoire de Biostatistique, Recherche Clinique et Epidémiologie (LBRCE), département de santé publique, Faculté de médecine et de pharmacie, Université Mohammed V Souissi, Rabat, Maroc

**Keywords:** Traumatisme crânien grave, coagulopathie, facteurs pronostiques, mortalité, Severe head trauma, coagulopathy, prognostic factors, mortality

## Abstract

**Introduction:**

Le but de ce travail était d'étudier la mortalité et les facteurs pronostiques de la Coagulopathie Traumatique (CoT) aigue précoce.

**Méthodes:**

Il s'agissait d'une étude prospective sur 21 mois, incluant tous les traumatisés crâniens graves > 16 ans. Les données clinico-démographiques, biologiques et radiologiques ont été collectées à l'admission. La CoT a été définie par une thrombopénie < 120 000 /mm^3^ t/ou un Taux de Prothrombine (TP) < 70% et/ou un International Normalized Ratio (INR) > 1,3 et/ou un Temps de Céphaline Activé (TCA) > 40 sec.

**Résultats:**

Nous avons colligé 69 patients ayant une CoT avec une médiane d'âge à 29 ans avec un intervalle interquartile à (23-41), et une prédominance masculine dans 87% des cas (60 patients). L'ISS médian à 56 avec un intervalle interquartile à (42-75). La mortalité était de 52,2% des cas (36 patients) suite à une souffrance cérébrale dans 74,3% des cas (26 patients). La durée médiane de séjour était de 10 jours avec un intervalle interquartile à (5-19). Les facteurs associés à la mortalité en analyse univariée étaient: le passager de véhicule, le délai de prise en charge, l'hématome sous dural aigu, l'AIS tête et l'ISS à l'admission; alors qu'en analyse multivariée c'étaient le délai de prise en charge (OR: 1,01; IC à 95%: 1,001-1,02) et l'ISS à l'admission (OR: 1,1; IC à 95%: 1,04-1,15) étaient des facteurs pronostiques indépendants.

**Conclusion:**

L'amélioration du pronostic de ces patients repose essentiellement sur un développement de la médecine pré-hospitalière.

## Introduction

De définition non consensuelle, la coagulopathie au cours des traumatismes crâniens (TC) est très fréquente, elle varie selon les séries et peut atteindre 100% des cas; Chiaretti et al [1] reportaient un taux de 10%, alors que Kuo et al [2] reportaient 66% chez les survivants des TC et 100% chez des TC décédés. La coagulopathie traumatique (CoT) a été publiée, pour la première fois, par Penick et McLendon en 1960 chez un nouveau-né traumatisé crânien [3]. L'hypothèse physiopathologique la plus plausible est l'activation de la coagulation par la libération d'activateurs de la coagulation par le cerveau lésé qui est riche en facteur tissulaire (thromboplastine) [4]; sans oublier de noter que le syndrome inflammatoire de réponse systématique post-traumatique peut aussi participer à l'activation de la coagulation [5]. En plus de ces conditions, récemment Hess et al [6] suggéraient que le traumatisme tissulaire, l'état de choc, l'hémodilution, l'hypothermie, l'acidose et l'inflammation contribuent à l'initiation de la COT. Au total, la CoT est due à des lésions tissulaires avec consommation des facteurs de la coagulation provoquant ainsi une coagulopathie endogène, et elle est accélérée par l'hypothermie, l'acidose métabolique et l'hémodilution [7]. Et plus précisément, Wohlauer et al suggéraient qu'une dysfonction plaquettaire est un biomarqueur précoce et sensible de la survenue d′une coagulopathie traumatique [8]. La CoT est considérée comme un facteur indépendant de mauvais pronostic au cours des traumatismes crâniens [9–12] et à notre connaissance, aucun travail qui étudiait ce phénomène dans notre contexte marocain n'a été publié. L'objectif de notre enquête était d'étudier la mortalité et les facteurs pronostiques de la CoT aigue précoce suite à un traumatisme crânien.

## Méthodes

Nous avons mené une étude prospective de Janvier 2011 au Septembre 2012 (21 mois), incluant tous les traumatisés crâniens graves > 16 ans ayant nécessité l'hospitalisation en réanimation du centre hospitalier universitaire de Marrakech-Maroc. Ont été exclut, les patients ayant une pathologie neurologique préexistante, une pathologie ou un traitement en cours affectant la coagulation et les traumatisés avec une atteinte orbitaire rendant difficile la surveillance des pupilles. La prise en charge médicale comportait un contrôle des voies aériennes supérieures (oxygénation et ventilation mécanique), une préservation de l'état hémodynamique (remplissage prudent, amines vasopressives si besoin, transfusion sanguine ayant comme objectifs une Hb ≥ 10 g/dl, des plaquettes ≥100 000 /mm^3^ et un TP ≥ 50%), une intervention neurochirurgicale si nécessaire, une sédation-analgésie, une prévention épileptique et une prévention des autres ACSOS (agressions cérébrales systémiques d'origine secondaire). Les données démographiques et cliniques ont été collectées à l'admission: âge, sexe, antécédents pathologiques, délai de prise en charge, mécanisme et nature du traumatisme, pouls, pression artérielle, température, fréquence respiratoire, SpO_2_ , les scores de gravité: Glasgow Coma Scale (GCS) [13], Abbreviated Injury Scale (AIS) [14] et Injury Severity Score (ISS) [14, 15], le durée de séjour en réanimation et l'évolution. Les paramètres biologiques ont été collectés aussi à l'admission puis quotidiennement jusqu'à la stabilisation des patients: hématocrite (%), taux des plaquettes (/mm^3^), taux de prothrombine (%) (TP), international normalized ratio (INR), temps de céphaline activé (TCA) (sec) et glycémie (g/l). Le scanner cérébral a été réalisé chez tous les TC et interprété par deux radiologues. Les mesures de prise en charge ont été consignées: remplissage à l'admission, nécessité des catécholamines, transfusion à l'admission et le recours à la chirurgie en urgence. La CoT a été définie par une thrombopénie < 120 000 /mm^3^ et/ou un TP < 70% et/ou un INR > 1,3 et/ou un TCA > 40 sec.

Les variables qualitatives ont été exprimées en effectifs et en pourcentages et comparées par le test Khi-deux. Les variables quantitatives ont été exprimées en moyenne ± DS ou en médiane avec interquartiles et comparées par le test-t Student ou le test de Mann Whitney. La normalité des variables quantitatives a été vérifiée par le test de Kolmogorov Smirnov. La régression logistique simple a été utilisée pour l'analyse univariée et la régression logistique multiple pour l'analyse multivariée. Un p < 0,05 a été considéré comme statistiquement significatif. Nous avons utilisé le logiciel SPSS version 13.0 pour Windows pour l'étude statistique.

## Résultats

Durant la période de l'étude, 109 patients ont été admis pour TC (16,26% des 670 admissions dans notre formation). Parmi ces 109 patients, 69 patients (63,3%) ont présenté une CoT. Le Tableau 1 résumait les caractéristiques cliniques, biologiques, thérapeutiques et évolutives. La médiane de l'âge était à 29 ans avec un intervalle interquartile à (23-41), et avec une prédominance masculine dans 87% des cas (60 patients). L'ISS médian à 56 avec un intervalle interquartile à (42-75). L'accident de la voie publique était le mécanisme prédominant dans 94,1% des cas. La mortalité était de 52,2% des cas (36 patients) suite à une souffrance cérébrale dans 74,3% des cas (26 patients). La durée médiane de séjour était de 10 jours avec un intervalle interquartile à (5-19). Les facteurs de mortalité en analyse univariée (Tableau 2) étaient: le passager d'un véhicule à 4 roues, le délai de prise en charge, l'hématome sous dural aigu, l'AIS tête et l'ISS à l'admission; alors qu'à l'analyse multivariée: le délai de prise en charge (OR: 1,01; IC95%: 1,001-1,02; p = 0,02) et l'ISS à l'admission (OR: 1,1; IC95%: 1,04-1,15; p = 0,001) étaient des facteurs pronostiques indépendants (Tableau 3).

**Tableau 1:** Caractéristiques démographiques, cliniques, biologiques, radiologiques et évolutives des patients ayant une coagulopathie traumatique aigue précoce

	Patients
Age; année, mediane [quartiles]	29 [23-41,5]
Sexe ratio (H/F)	60/9
Délai de prise en charge; min, mediane (quartiles)	120 [60-180]
GCS; (moyenne ± DS)	9 ± 3
Pupilles anormales ; n (%)	20 (30)
PAS; mmHg,mediane [quartiles]	120 [100-129]
PAD; mmHg,mediane [quartiles]	70 [60-80]
SpO_2_ < 95% ; n (%)	8 (12)
Remplissage; litres, (moyenne ± DS)	1,7 ± 1,15
Catécholamines ; n(%)	12 (18)
Transfusion; n(%)	33 (49)
Chirurgie en urgence; n(%)	19 (29)
Hématome extradural; n(%)	13 (19)
Hématome sous dural; n(%)	23 (33)
Hématome intraparenchymateux; n(%)	4 (6)
Hémorragie intraventriculaire; n(%)	16 (23)
Contusion; n(%)	36 (52)
Pneumocéphalie; n(%)	5 (7)
Déviation de la ligne médiane; n(%)	17 (25)
AIS tête ; (moyenne ± DS)	4,8 ± 1
ISS; mediane [quartiles]	54 [42-75]
Hématocrite à J1; mg/dl, (moyenne ± DS)	32 ± 7,6
Plaquettes à J1; .10^3^/mm^3^, (moyenne ± DS)	181,57 ± 89,7
Taux prothrombine à J1;%, (moyenne ± DS)	56 ± 15
INR à J1; (moyenne ± DS)	1,8 ± 0,8
TCA à J1; sec, (moyenne ± DS)	33 ± 11
Glycémie à J1; g/l mediane [quartiles]	1,36 [1,14-2]
Durée d'hospitalisation; jours, mediane [quartiles]	10 [5-18]
Décès; n(%)	36 (52,2)

**GCS**, Glasgow Coma Scale ; **PAS**, Pression Artérielle Systolique ; **PAD**, Pression Artérielle Diastolique ; **AIS**, Abbreviated Injury Score ; **ISS**, Injury Severity Score ;**INR**, international normalized ratio ; **TCA**, Temps Céphaline Activée.

**Tableau 2:** Analyse univariée des paramètres des patients survivants vs décédés ayant une coagulopathie traumatique aigue précoce (Khi-2 et t-test de Student)

	Analyse univariée		
	Survivant N = 33	Décédé N = 36	p
Age; année, mediane [quartiles]	32,9 ± 13	33,7 ± 14	0,8
Sexe ratio (H/F)	4,5	11	0,2
Passager d'un véhicule à 4 roues (%)	30	70	0,04
Délai de prise en charge; min, mediane (quartiles)	120 (60;120)	180 (120;240)	0,004
GCS; (moyenne ± DS)	10 ± 3	8 ± 2	0,07
Pupilles anormales; n (%)	8 (25)	12 (35)	0,3
PAS; mmHg,mediane [quartiles]	114 ± 24	112 ± 25	0,7
Remplissage; litres, (moyenne ± DS)	1,42 ± 1	1,9 ± 1,2	0,06
Catécholamines; n(%)	3 (9)	9 (25)	0,07
Transfusion; n(%)	13 (40)	20 (57)	0,17
Transfusion culot globulaire; unité, (moyenne ± DS)	3 ± 1	4 ± 1	0,057
Transfusion PFC ; unité, (moyenne ± DS)	5 ± 1	6 ± 1	0,5
Transfusion des plaquettes; unité, (moyenne ± DS)	4 ± 2	5 ± 2	0,8
Hématome extradural; n(%)	6 (18)	7 (19)	0,9
Hématome sous dural (%)	31	69	0,04
Hématome intraparenchymateux; n(%)	1 (3)	3 (8)	0,3
Contusion; n(%)	14 (42)	22 (61)	0,12
Pneumocéphalie; n(%)	3 (9)	2 (5)	0,6
AIS tête; (moyenne ± DS)	4,3 ± 1,04	5,2 ± 0,86	<0,001
ISS; mediane (quartiles)	45 (35;57)	75 (52;75)	<0,001
ISS > 30 (%)	43	57	0,03
Hématocrite à J1; mg/dl, (moyenne ± DS)	31,5 ± 7	33 ± 8	0,4
INR à J1; (moyenne ± DS)	1,9 ± 1,06	1,6 ± 0,53	0,3
Glycémie à J1; g/l mediane [quartiles]	1,54 ± 0,6	1,57 ± 0,6	0,8
Durée d'hospitalisation; jours, mediane [quartiles]	11 (6;20)	10 (5;18)	0,3

**GCS**, Glasgow Coma Scale; **PAS**, Pression Artérielle Systolique; **PFC**, Plasma Frais Congelé ;**AIS**, Abbreviated Injury Score ; **ISS**, Injury Severity Score ; **INR**, international normalized ratio.

**Tableau 3:** Facteurs pronostiques des patients ayant une coagulopathie traumatique par régression logistique simple et multiple.

	Régression logistique simple			Régression logistique multiple		
	OR	p	IC 95%	OR	p	IC 95%
Passager	0,33	0,53	0,11-1,01	-	-	-
Délai de prise en charge	1,01	0,01	1,002-1,02	1,01	0,02	1,001-1,02
Hématome sous dural	0,34	0,045	0,12-0,97	-	-	-
AIS tête	3,06	0,002	1,53-6,11	1,75	0,2	0,70-4,33
ISS	1,08	<0,001	1,04-1,13	1,1	0,001	1,04-1,15
INR à l'admission	0,63	0,22	0,3-1,32	-	-	-

**AIS**, Abbreviated Injury Score ; **ISS**, Injury Severity Score ; **INR**, international normalized ratio.

## Discussion

Dans notre étude, nous avons observé un taux élevé de CoT chez les TC dépassant les 63% et le décès est survenu chez plus de la moitié parmi eux. Le retard de prise en charge était associé avec une mortalité élevée. Tout en sachant qu'au Maroc le transport des blessés est sous la responsabilité de la protection civile et qui est parfois dépassée par la demande de plus en plus accrue; et l'instauration de la médecine pré-hospitalière est en cours de développement sur le territoire marocain. La CoT est fréquente et constitue un facteur de sévérité à l'admission des patients, voire un facteur aggravant au cours de l'hospitalisation [16] ce qui concordait avec nos résultats. La CoT survient très précocement sur le lieu du traumatisme avant même la mise en route d'une perfusion, et elle est liée plus à la gravité du traumatisme [17]. Le délai de prise en charge a été dégagé comme un facteur pronostique indépendant dans notre étude; du fait que c'est la protection civile qui s'occupe du ramassage et du transport des victimes vers les hôpitaux. Cliniquement, le risque d'une évolution défavorable (Glasgow Outcome Scale à 1-3) en présence d'une CoT est estimé à un odds ratio de 36.3 (95%CI: 8.7-70.5), et de décès à 9.4 (IC 95%: 7,6-11,6) [18], en plus le GCS bas est défini comme un prédicteur indépendant de mortalité [19]. Biologiquement, Olson et al [18] et Selladurai et al [20] ont démontré que le produit de dégradation de la fibrine (PDF) prédit un mauvais pronostic indépendamment des autres variables, et le pronostic s'aggrave au fur et à mesure de l'augmentation du PDF. Le temps de la prothrombine a été révélé aussi comme un facteur pronostique indépendant par l'étude IMPACT (International Mission for Prognosis And Clinical Trial) [21]. En plus du TP, Salehpour et al ont conclu que l'INR et le TCA peuvent être utilisés comme des facteurs de prédiction de devenir et du pronostic des TC graves [22]. Les scores de gravité et notamment l'ISS a été décrit d'être un facteur indépendant de mortalité au cours des TC isolés [23].

A coté de notre modeste échantillon, le manque d'exploration de certains paramètres biologiques ont fait les limites de notre travail, qui malgré cela, il nous a permis d'estimer l'ampleur de la CoT dans notre contexte ainsi que la mortalité et quelques facteurs pronostiques de ce phénomène.

## Conclusion

Le retard de prise en charge et la gravité du traumatisme (ISS élevé) étaient associés à une mortalité élevée. L'amélioration du pronostic de la coagulopathie traumatique précoce impose le développement de la médecine pré-hospitalière, en plus d'une surveillance récurrente de la coagulation pour détecter précocement les anomalies de l'hémostase.
